# An Ethylene-responsive Factor *BpERF11* Negatively Modulates Salt and Osmotic Tolerance in *Betula platyphylla*

**DOI:** 10.1038/srep23085

**Published:** 2016-03-16

**Authors:** Wenhui Zhang, Guiyan Yang, Dan Mu, Hongyan Li, Dandan Zang, Hongyun Xu, Xuezhong Zou, Yucheng Wang

**Affiliations:** 1 State Key Laboratory of Forest Genetics and Tree Breeding, Northeast Forestry University, 150040 Harbin, China; 2 Agronomy College, Heilongjiang Bayi Agricultural University, 163319 Daqing, China; 3 Liaoning Forestry Vocation-Technical College, 110101 Shenyang, China

## Abstract

Ethylene responsive factors (ERFs) play important roles in the abiotic stress; however, only a few ERF genes from woody plants have been functionally characterized. In the present study, an ERF gene from *Betula platyphylla* (birch), *BpERF11*, was functionally characterized in response to abiotic stress. BpERF11 is a nuclear protein, which could specifically bind to GCC boxes and DRE motifs. *BpERF11*-overexpressing and *BpERF11* RNA interference (RNAi) knockdown plants were generated for gain- and loss-of-function analysis. *BpERF11* negatively regulates resistance to salt and severe osmotic stress, and the transgenic birch plants overexpressing *BpERF11* shows increased electrolyte leakage and malondialdehyde (MDA) contents. *BpERF11* inhibits the expression of an *AtMYB61* homologous gene, resulting in increased stomatal aperture, which elevated the transpiration rate. Furthermore, *BpERF11* downregulates the expression of *P5CS*, *SOD* and *POD* genes, but upregulates the expression of *PRODH* and *P5CDH*, which results in reduced proline levels and increased reactive oxygen species (ROS) accumulation. *BpERF11* also significantly inhibits the expression of *LEA* and dehydrin genes that involve in abiotic stress tolerance. Therefore, *BpERF11* serves as a transcription factor that negatively regulates salt and severe osmotic tolerance by modulating various physiological processes.

Plants are usually subjected to various abiotic challenges from the environment, such as salt, drought or extreme temperature. Saline soil is continuous increasing because of modern agricultural practices. It is estimated that by the year 2050, more than 50% of all cultivated lands may suffer serious salinization^1^. Drought is widespread in many regions and is expected to increase further^2^. Both drought and salt stress severely damage the survival and development of plants, resulting in limited the growth, development and productivity^3^. Plants have evolved complicated systems to protect themselves against different abiotic stresses. In plants, transcription factors (TFs) play key roles in gene expression regulation in response to abiotic stress. Among the TF families, AP2/ERF, bZIP, WRKY, MYB and NAC families play main roles in gene expression regulatory networks in response to abiotic stresses^4^,^5^,^6^. ERFs are unique to plants, and are involved in the regulation of abiotic stress responses and provide exciting potential for engineering abiotic stress-tolerant plants^7^,^8^,^9^,^10^,^11^. ERF is a large family, with 157, 147 and 148 members in rice, *Arabidopsis* and soybean genomes, respectively^12^,^13^,^14^.

Buttner *et al.*^15^ showed that ERF can activate or inhibit the transcription of genes that have GCC box (AGCCGCC) in their promoters, dependently or independently of the ethylene signal pathway. Additionally, some ERFs also can bind to a CRT/DRE (A/GCCGAC) motif to regulate the expression of genes in response to biotic or abiotic stresses^16^,^17^,^18^,^19^.

ERFs are involved in various biological processes, such as primary and secondary metabolism, flowers and seeds development, roots development, especially they could modulate multiple responses to abiotic stresses^20^,^21^,^22^,^23^. For instance, wheat TaERF1 binds to GCC boxes and CRT/DRE motifs, and its overexpression activated the expression of pathogenesis-related (*PR*) and cold-regulated/responsive to dehydration (*COR/RD*) genes, which are related to stress tolerance to drought, cold and salt stresses^24^. Mishra *et al.*^25^ isolated an AP2/ERF from *Papaver somniferum* (PsAP2) that could bind to DRE and GCC box motifs, and *PsAP2* overexpressing transgenic tobacco plants exhibited increased tolerance to both abiotic and biotic stresses. Gao^26^ cloned the *TERF1* gene from tomato, and transferred it into rice. The transgenic rice plants displayed improved salt and drought tolerance by not only improving proline accumulation, but also reducing water loss. Additionally, TERF1 induces the expression of LIP5, Wcor4131, OsPrx and OsABA2 by binding to GCC boxes or DRE/CRT motifs. *AtERF71/HRE2* from *Arabidopsis* is significantly induced by NaCl, mannitol and abscisic acid (ABA). Furthermore, plants overexpressing *AtERF71* showed improved tolerance to salt and mannitol, as well as flooding and methyl viologen (MV) stresses, and showed decreased reactive oxygen species (ROS) accumulation under high salt stress^27^. Overexpression of *GmERF7* significantly improved salt tolerance in transgenic tobacco, and the chlorophyll and carbohydrate contents increased in the transgenic plants, meanwhile, the MDA content was reduced^28^. However, some ERF genes negatively regulate stress tolerance. For instance, the expression *AtERF4* is induced by ethylene (ET), methyl jasmonate (MeJA) or ABA, and its overexpression in transgenic *Arabidopsis* decreased sensitivity to ABA and made the plants hypersensitive to NaCl. Moreover, the expression of several stress-responsive genes decreased in transgenic plants compared with wild-type (WT) plants^29^. AtERF7 binds to GCC boxes and serves as a repressor of gene transcription. *Arabidopsis* plants overexpressing *AtERF7* displayed reduced sensitivity of guard cells to ABA and increased transpirational water loss, suggesting that *AtERF7* is important in ABA responses and is involved in transcriptional repression^30^. OsERF922 binds specifically to GCC boxes to act as a transcriptional activator, and *OsERF922*-overexpressing plants showed reduced salt stress tolerance, with an increased Na^+^/K^+^ ratio in transgenic plants^31^. Potato StERF3 is localized to the nucleus and binds to the GCC box element. *StERF3-*overexpression plants negatively regulated resistance to salt tolerance in potato, however, *StERF3* RNAi knockdown plants showed elevated salt tolerance accompanied by the activation of defense related genes (*PR1, NPR1* and *WRKY1*)^32^. In addition, *ERFs* might play an important role in fine-tune the stress response. For instance, *Arabidopsis ERF6* inhibits leaf growth and induces the expression of stress tolerance genes and *ERF11*. However, the induced *ERF11* in turn counteracts the action of *ERF6* by repressing some of the *ERF6*-induced genes, and *ERF11* overexpression could suppress the extreme dwarfism caused by *ERF6*. These results demonstrated that *ERF11* counteracts *ERF6* to keep a balance between stress tolerance and growth in plants^33^.

In the current study, we cloned an ERF gene, *BpERF11*, from *Betula platyphylla* (white birch). To investigate its roles in response to salt and severe osmotic stress tolerance, we studied the expression of *BpERF11* in birches exposed to salt and severe osmotic stress, and studied its binding to DRE and GCC box motifs. Furthermore, transgenic birches either overexpressing or knocked down for *BpERF11* were generated for gain- and loss-of function analysis. The physiological changes involved in stress tolerance and the expression of genes were studied in these transgenic birch lines. The mechanism of stress tolerance mediated by *BpERF11* was revealed in this study. Our study increases our understanding of the function of *BpERF11* in response to abiotic stress.

## Results

### Cloning and sequence analysis of *BpERF11*

The cDNA sequence of *BpERF11* was identified from the transcriptomes of *B. platyphylla*^34^. The coding sequence (CDS) of *BpERF11* is 444 bp in length and was predicted to encode a 147 amino acid protein with molecular mass of 36.48 kDa. Multiple sequence alignments and phylogenetic analysis revealed that BpERF11 shares highest amino acid sequence homology to AT3G50260.1 (AtERF011) (Fig. S1A,B). This result indicated that *BpERF11* belongs to the A-5 group of the ERF/AP2 transcription factor family, which has a highly conserved AP2 domain, and the amino acid residues at positions 14 and 19 in the conserved domain were valine and glutamic. The sequence of *BpERF11* had been deposited in GenBank with the accession number of KT601336.

### Expression of *BpERF11* is induced by NaCl, mannitol and PEG treatments

**Figure 1:**
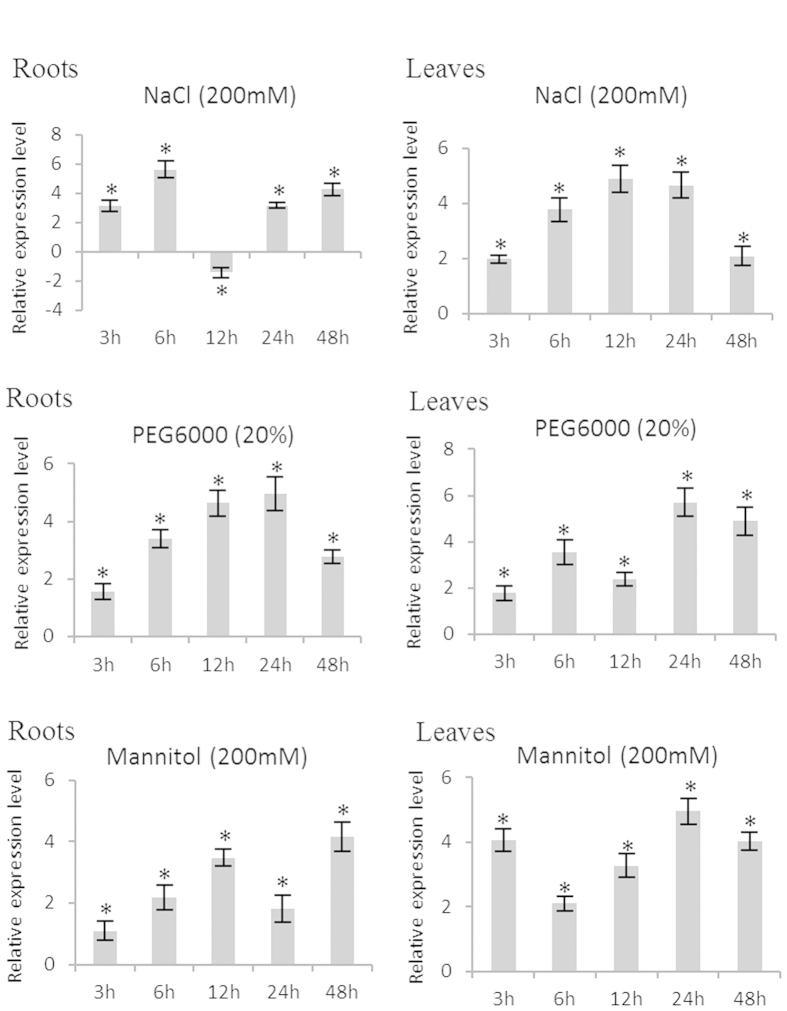
Expression profiles of *BpERF11* in root and leaf tissues in response to NaCl, PEG and mannitol stress. The expression levels at each time point is normalized by the expression level in the samples treated with fresh water and harvested at the corresponding time point (control), and were log2 transformed. Three biological replications were conducted. The error bars represent the standard deviation (S. D.). Asterisk indicates significant difference between treatments and controls (P < 0.05).

To determine the potential functions of *BpERF11* in response to different abiotic stresses, the expression of *BpERF11* in birch roots and leaves under NaCl, mannitol and PEG stress conditions were determined. In roots, *BpERF11* was highly induced by PEG stress during the studied period. *BpERF11* was also significantly upregulated by mannitol and NaCl, especially at NaCl stress for 6 h, or mannitol stress for 12 and 48 h. In leaves, the expression of *BpERF11* was significantly induced by NaCl, mannitol and PEG stress. Additionally, *BpERF11* was highly expressed at NaCl stress for 6 to 24 h, but was highly induced by mannitol and PEG at stress for 24 to 48 h. These results indicated that *BpERF11* might play a role in salt and osmotic stress responses in birch plants (Fig. 1).

### Subcellular localization of BpERF11 protein

**Figure 2:**
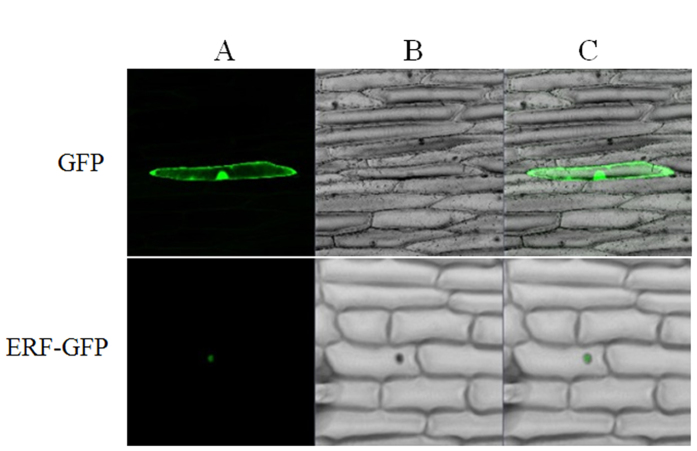
Subcellular localization analysis of BpERF11. (**A**) GFP fluorescence; (**B**) bright field; (**C**) merged image of bright field and fluorescence. ERF-GFP: 35S:ERF-GFP was transiently transformed into onion epidermal cells. GFP: 35S:GFP was transiently transformed into onion epidermal cells as a control.

To determine the subcellular localization of BpERF11 protein, the 35S: BpERF11-GFP and 35S:GFP constructs were introduced into onion epidermal cells using particle bombardment. The fluorescence signals in the inner epidermal cells were observed using confocal laser scanning microscopy. BpERF11-GFP fusion protein was visualized mainly in the nuclei, whereas the control (transformed with 35S:GFP) was observed throughout the cells (Fig. 2). The results indicated that BpERF11 is a nuclear protein.

### BpERF11 specifically binds to GCC box and DRE motif

**Figure 3:**
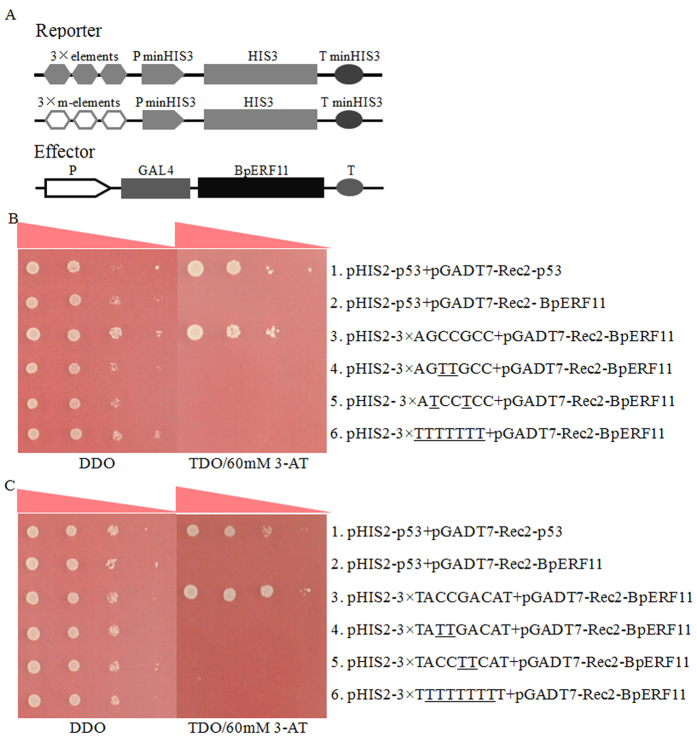
Analyses of the bindings of BpERF11 to the GCC box and DRE motifs. Three tandem copies of the GCC box, DRE or their mutants were cloned separately into the pHIS2 vector, and transformed into yeast Y187 together with pGADT7-Rec2-*BpERF11*. The positive transformants were determined by spotting serial dilutions (1:1, 1:10, 1:100, 1:1000) of yeast onto SD/–His/–Leu/–Trp plates supplemented with 3-AT. (**A**) Diagram of reporter and effector vectors used in the Y1H analysis; (**B**,**C**) BpERF11 interacts with the GCC box (**B**) and DRE motif (**C**). Positive control (pHIS2-p53/pGADT7-Rec2-p53); Negative control (pHIS2-p53/pGADT7-Rec2-*BpERF11*).

To determine whether BpERF11 also could bind to GCC boxes and DRE motifs, Y1H analysis was carried out. The pHIS2 vectors containing GCC box, DRE motifs or their mutants were co-transformed with pGADT7-rec2 effector vectors harboring *BpERF11* into Y187 yeast cells to test their interaction. BpERF11 could specifically bind to GCC boxes and DRE motifs, but failed to bind to all the mutated GCC boxes and DRE motifs (Fig. 3B,C), indicating that BpERF11 specifically binds to GCC boxes and DRE motifs.

### Expression of *BpERF11* in transformed lines

To study the biological role of *BpERF11* in stress responses, we generated transgenic birch plants that overexpressed *BpERF11* (OE) or comprised RNAi-silencing of *BpERF11* (Ri). Nine independent OE lines and seven Ri lines were generated. The expression of *BpERF11* in the transgenic lines was studied using qRT-PCR. The expression of *BpERF11* was significantly upregulated in the OE lines compared with WT plants, OE3 and OE6 showed the highest levels, and were selected for further study. Expression levels of *BpERF11* were downregulated in all Ri lines and Ri2 and Ri3 lines were selected for further experiments due to their lowest expression levels (Fig. S2).

### *BpERF11* negatively controls salt and severe osmotic stress tolerance

**Figure 4:**
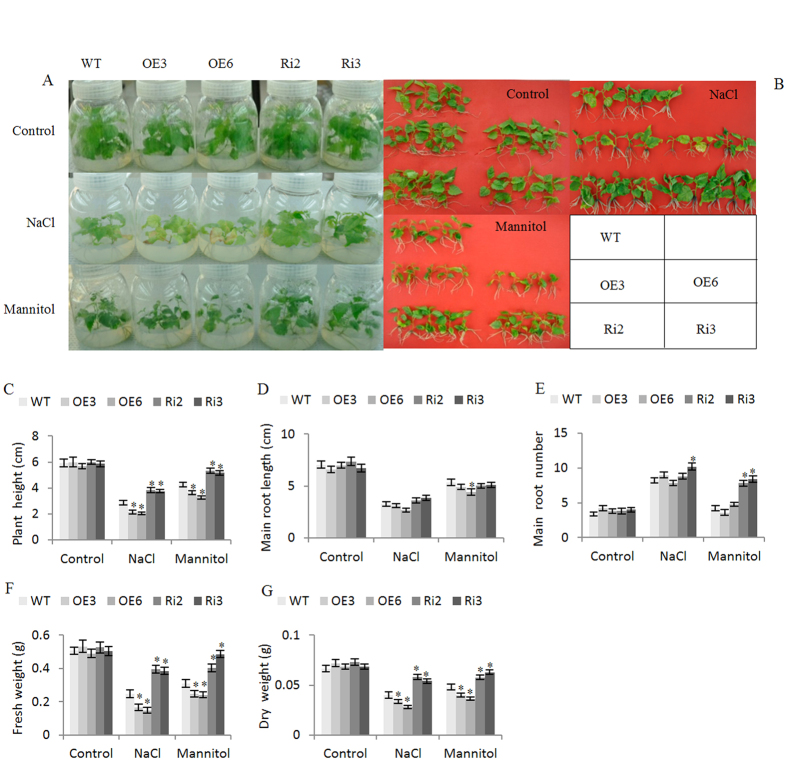
Analysis of salt and mannitol stress tolerance. (**A,B**) Birch shoots (about 1 cm in length) of WT, OE and Ri lines were grown on WPM (normal conditions), or WPM medium supplied with 50 mM NaCl or 80 mM Mannitol for 4-weeks, and their growth and phenotype were recorded; (**C–G**) Plant height (**C**), main root length (**D**), root number (**E**), fresh weight (**F**) and dry weight (**G**). Three independent biological replications were conducted. The error bars represent the standard deviation (S. D.). Asterisk indicates significant difference between OE lines and WT or between Ri lines and WT (P < 0.05).

For the abiotic stress tolerance assay, the transgenic lines OE3, OE6, Ri2, Ri3 and WT plants grown in WPM medium were treated with 50 mM NaCl or 80 mM mannitol for 4 weeks. Under normal growth conditions, there was no obvious difference in phenotype and plant height, root number, root length, fresh and dry weight among the WT, OE and Ri lines (Fig. 4A–G). However, when exposed to salt or mannitol conditions, the plant heights, fresh and dry weight in both Ri lines were all the highest, followed by the WT plants; the OE lines displayed lowest heights, and fresh and dry weights (Fig. 4C,F,G). These results indicated that *BpERF11* negatively regulates salt and severe osmotic stress tolerance.

### Analysis of chlorophyll contents and electrolyte leakage

OE, Ri and WT birch plantlets of similar sizes grown in a mixture of turf peat and sand were treated with 150 mM NaCl or 200 mM mannitol for 3 d, and their chlorophyll contents and electrolyte leakage rates were analyzed. There were no significant differences in chlorophyll contents among the OE, Ri and WT lines under normal conditions. Under salt or mannitol stress conditions, the chlorophyll contents were reduced in all the studied lines. However, both OE lines exhibited lowest chlorophyll levels, and the Ri lines displayed highest levels (Fig. S3A). Electrolyte leakage analysis showed that the OE, WT and Ri lines had similar electrolyte leakage rates under normal conditions. However, the Ri lines displayed significantly decreased electrolyte leakage rates and OE lines had significantly increased electrolyte leakage rates compared with the WT plants under salt or mannitol stress conditions (Fig. S3B). Consistently, we also measured the damage to cell membranes using Evans blue staining (Fig. S3C). The results showed no difference among these lines under normal growth conditions; however, the OE lines suffered higher cell death than the WT plants, and the Ri lines displayed lower cell death rates compared with the WT under salt or severe osmotic stress treatment.

### ROS scavenging analyses

**Figure 5:**
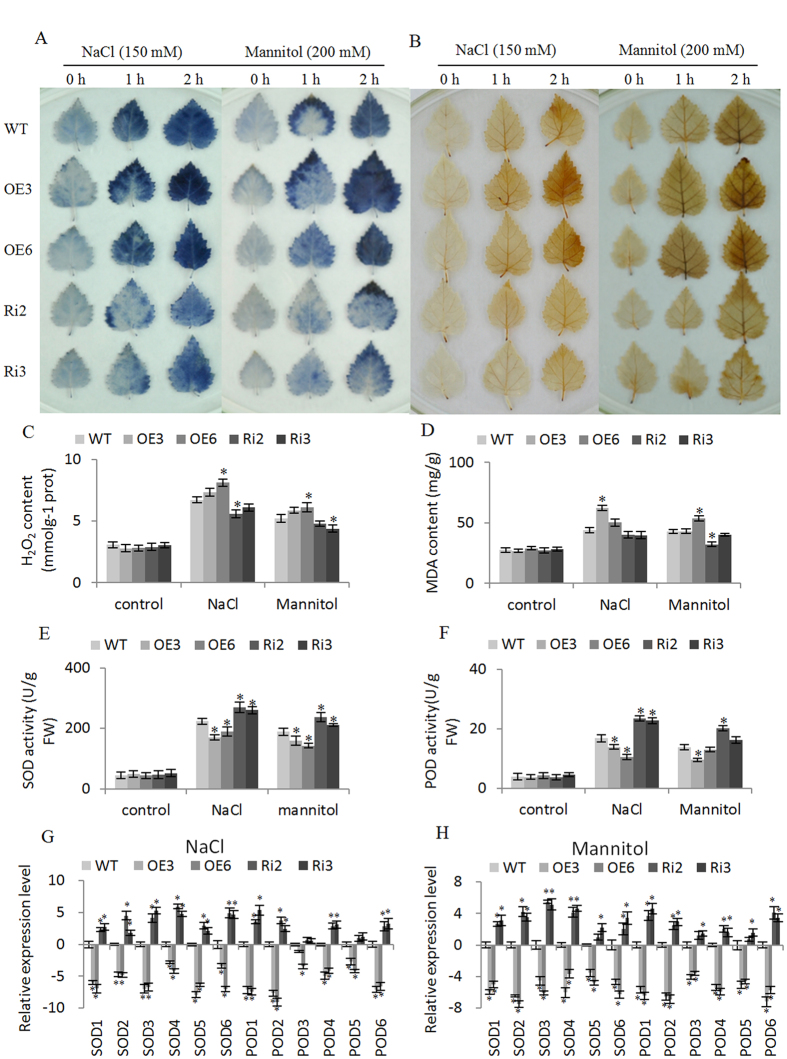
ROS scavenging analyses. (**A,B**) diaminobenzidine (DAB) (**A**) and nitroblue tetrazolium (NBT) (**B**) staining to reveal H_2_O_2_ and O_2_^−^ accumulation, respectively. The birches grown on WPM medium were treated with 150 mM NaCl or 200 mM Mannitol for 1 or 2 h, and then leaves were detached for DAB and NBT staining; leaves grown on WPM were the control; (**C,D**) H_2_O_2_ content (**C**) and MDA content (**D**) analysis; (**E,F**) SOD (**E**) and POD (**F**) activity analysis; (**G**) the expression analysis of *SOD* and *POD* genes in OE, WT and Ri lines under NaCl stress; (**H**) the expression analysis of *SOD* and *POD* genes in OE, WT and Ri lines under mannitol stress. Three biological replications were conducted. The error bars represent the standard deviation (S. D.). Asterisk indicates significant difference between OE lines and WT or between Ri lines and WT (P < 0.05).

The H_2_O_2_ and O^2−^ levels were examined by DAB and NBT staining, respectively. There were no differences in NBT and DAB staining among all the studied lines under normal growth conditions (Fig. 5A,B). However, when exposed to salt or osmotic stress, both the Ri lines showed obviously reduced H_2_O_2_ and O^2−^ levels compared with the WT plants, and the OE3 and OE6 lines had higher H_2_O_2_ and O^2−^ levels than the WT (Fig. 5A,B). Consistent with the DAB staining, H_2_O_2_ measurement also showed that both OE lines showed the highest H_2_O_2_ levels, followed by the WT; the Ri lines had the lowest level under NaCl and mannitol stress conditions. All the lines had similar H_2_O_2_ level without stress (Fig. 5C). Additionally, under salt or osmotic stress conditions, the MDA contents in OE lines were highest, followed by the WT; both Ri lines had the lowest levels. The MDA levels were similar among these lines under normal conditions (Fig. 5D). These results indicated that membrane lipid peroxidation was negatively regulated by *BpERF11*.

As the H_2_O_2_ and O^2−^ levels were significantly altered, we further examined SOD and POD activity in each studied line (Fig. 5E,F). Under normal conditions, all the studied lines showed similar SOD and POD activities. Under NaCl or mannitol treatment conditions, SOD and POD activities increased in all lines; however, both OE3 and OE6 lines had lower SOD and POD activities than the WT, and the Ri lines showed significantly higher SOD and POD activities than the WT plants (Fig. 5E,F).

We next examined the expression of *SOD* and *POD* genes to determine whether the altered SOD and POD activities were caused altered expression of *SOD* and *POD* genes. The results showed that the expression of all the studied *SOD* and *POD* genes were highest in the Ri lines and lowest in the OE lines, indicating that *BpERF11* downregulates the expression of *SOD* and *POD* genes (Fig. 5G,H).

### Proline biosynthesis analysis

**Figure 6:**
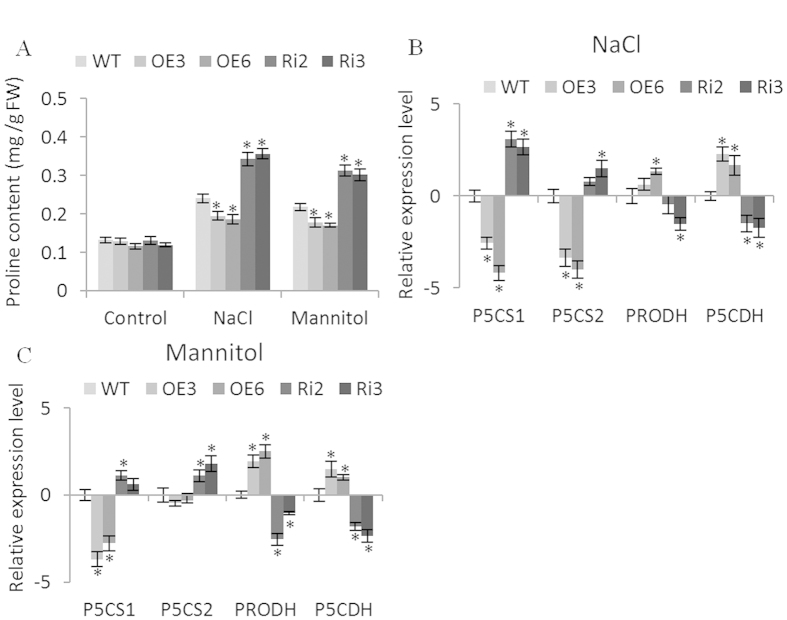
Biosynthesis of proline mediated by *BpERF11*. **(A)** Proline content analysis under normal, salt or mannitol treatment conditions; (**B,C**) The expression level of proline biosynthesis-related genes (*BpP5CS1*and *BpP5CS2*) and proline degradation-related genes (*BpPRODH* and *BpP5CDH*) in WT, OE and Ri lines under NaCl (**B**) or mannitol (**C**) treatment conditions. Three biological replications were conducted. The error bars represent the standard deviation (S. D.). Asterisk shows significant difference between OE lines and WT or between SE lines and WT (P < 0.05).

To study whether the altered stress tolerance mediated by *BpERF11* is due to a change in osmotic potential, we determined the proline contents. There was no significant difference among the WT, OE and Ri lines under normal conditions. However, under NaCl or mannitol stress conditions, the Ri lines had significantly higher proline levels than the WT plants, and the OE lines had the lowest proline levels (Fig. 6A). We further analyzed the expression of genes related to proline biosynthesis, including two proline biosynthesis genes, *BpP5CS1* and *BpP5CS2*, which are homologs of *AtP5CS1* (AT2G39800) and *AtP5CS2* (AT3G55610), respectively, from Arabidopsis plants; and proline degradation genes *BpPRODH* and *BpP5CDH*, which are homologs of Arabidopsis *AtPRODH* (AT3G30775) and *AtP5CDH* (AT5G62530), respectively. The expression levels of *BpP5CS1* and *BpP5CS2* were both significantly increased in the Ri lines compared with WT plants, but showed significant lower expression levels in OE lines compared with WT plants under NaCl or mannitol stress (Fig. 6B,C). Meanwhile, proline degradation genes, *BpPRODH* and *BpP5CDH*, were greatly downregulated in Ri lines, but greatly upregulated in the OE lines compared with WT plants (Fig. 6B,C). These results indicated that the biosynthesis of proline was highly inhibited, and the degradation of proline was enhanced in plants overexpressing *BpERF11*.

### Stomatal aperture and water loss rates analysis

**Figure 7:**
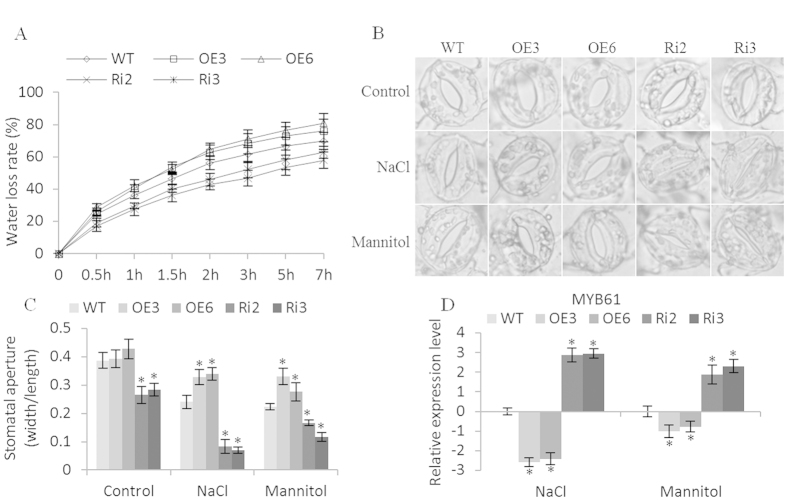
Water loss rate and stomatal aperture assays. (**A**) Water loss rate analysis. Leaves of 4-week-old birches from OE, WT and Ri lines were cut to measure water loss rate; (**B**,**C**) stomatal aperture analysis. Leaves of 4-weeks birches from OE, WT and Ri lines were treated with 50 mM NaCl or 100 mM mannitol for 2 h to measure stomatal aperture. (**D**) analysis of the expression of *BpMYB61* gene in OE, WT and Ri lines. The *BpMYB61* is homologous with *AtMYB61* (AT1G09540.1) from *Arabidopsis*. Three biological replications were conducted. The error bars represent the standard deviation (S. D.). Asterisk indicates significant difference between OE lines and WT or between SE lines and WT (P < 0.05).

We further studied whether transpiration rates were mediated by *BpERF11*. Compared with WT plants, Ri lines had significantly lower water loss rates, whereas both OE lines had increased water loss rates (Fig. 7A). As stomatal aperture is closely related to transpiration rates in plants, to determine whether the altered water loss rate was caused the changed stomatal aperture, we further measured the stomatal aperture. In Ri lines, the stomatal aperture (width/length) was significantly reduced compared with the WT, and the stomatal apertures in OE lines were significantly increased when exposed to salt and mannitol stress (Fig. 7B,C), suggesting that *BpERF11* negatively controls stomatal aperture to decrease water loss. As *AtMYB61* (AT1G09540) controls the stomatal aperture, we further studied the expression of *BpMYB61*, an *AtMYB61* homolog, in the OE, WT and Ri lines. The expression of *BpMYB61* was downregulated in the OE lines, but upregulated in the Ri lines (Fig. 7D), suggesting that the expression of *BpERF11* could downregulate the expression of *BpMYB61*.

### The expression of *LEAs* and *DHN* were downregulated by *BpERF11*

**Figure 8:**
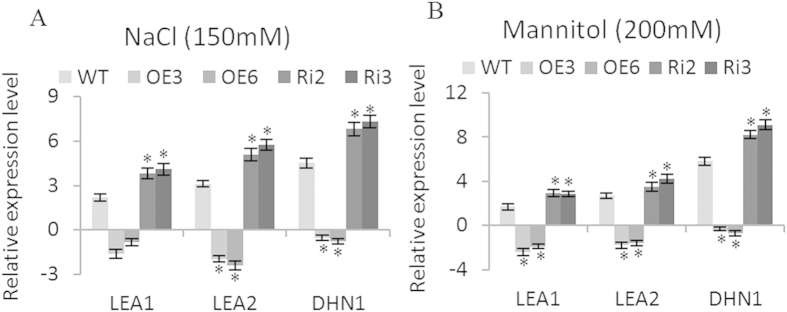
Analysis of *LEA* and dehydrin (*DHN*) genes regulation by *BpERF11*. *LEA1* and *LEA2* are homologous to AT5G66400 and AT1G52690, respectively, and *DHN1* is homologous to AT1G01470 from *Arabidopsis*. (**A**) The expression level of *LEA1*, *LEA2* and *DHN1* genes in OE, WT and Ri lines under NaCl treatment; (**B**) The expression level of *LEA1*, *LEA2* and *DHN1* genes in OE, WT and Ri lines under mannitol treatment. Three biological replications were conducted. Data are shown as the mean ± SD. Asterisks shows significant differences between OE lines and WT or between SE lines and WT (P < 0.05).

Two LEA genes (*BpLEA1*, *BpLEA2*) and one *DHN* gene (*BpDHN1*) from birch that are homologous to AT5G66400, AT1G52690 and AT1G01470 from *Arabidopsis*, respectively, and which play important roles in tolerance to salt and drought stresses were selected for expression analysis^35^,^36^,^37^. Under salt and severe osmotic stress conditions, *BpLEA1*, *BpLEA2* and *BpDHN1* were upregulated in the Ri lines with higher expression levels than in the WT; however, downregulated in the OE lines (Fig. 8A,B), indicating that *BpERF11* downregulates the expression of these genes.

## Discussion

In the present study, we cloned *BpERF11* from *B. platyphylla*. Previous studies showed that ERF members can interact with GCC boxes and DRE cis-elements^16^,^17^,^18^. Our results showed that BpERF11 is a nuclear protein that can also specifically bind to GCC boxes and DRE cis-elements (Fig. 3), indicating that it functions as a transcription factor in birch plants. Additionally, BpERF11 shares its highest amino acid sequence similarity with *AtERF011* (AT3G50260) (Fig. S1), suggesting that they might have similar functions. *BpERF11* is induced by salt and osmotic stress (Fig. 1), suggesting that it is involved in abiotic stress. However, the function of *AtERF011* in response to salt or severe osmotic stress has not been studied. In the present study, we functionally characterized *BpERF11*’*s* involvement in salt and severe osmotic stress tolerance.

### *BpERF11* negatively regulates salt and osmotic stress tolerance

We generated transgenic birch plants overexpressing *BpERF11* and RNAi-silenced *BpERF11* for gain- and loss-of-function analysis. Our results showed that transgenic plants overexpressing *BpERF11* displayed significantly decreased salt and severe osmotic tolerance (Fig. 4A–C,F,G); however, the knockdown of *BpERF11* in transgenic birch plants significantly improved salt and osmotic stress tolerance (Fig. 4A–C,F,G), suggesting that *BpERF11* negatively regulates abiotic stress tolerance.

### Overexpression of *BpERF11* increases stomatal aperture

The transpiration rate is very important for stress tolerance of plants, especially for water stress tolerance. The stomatal aperture plays a key role in controlling the transpiration rate. Our results showed that the expression of *BpERF11* in plants is negatively correlated with the transpiration rate (Fig. 7A); therefore, we further examined the stomatal aperture in OE, WT and Ri plants. The results showed that overexpression of *BpERF11* increased the stomatal aperture, whereas knockdown of *BpERF11* significantly decreased the stomatal aperture (Fig. 7B,C). In *Arabidopsis*, *AtMYB61* is involved in control of the closure of stomatal aperture^38^. To study whether *BpERF11* could regulate stomatal aperture-regulation genes, an *AtMYB61* (AT1G09540.1) homolog protein, *BpMYB61*, was studied. The expression of *BpMYB61* was strongly downregulated by *BpERF11* (Fig. 7B,C). These results demonstrated that *BpERF11* reduced the expression of *BpMYB61* to induce the opening of the stomatal aperture, which significantly increased the water loss rate, leading to sensitivity to salt and severe osmotic stress.

### *BpERF11* downregulates the expression of *LEA* and *DHN*

LEA family proteins act as molecular chaperones or shields to prevent irreversible protein aggregation, and can also stabilize the membrane by replacing water or inducing preferential hydration during desiccation conditions^39^. Among the group 2 *LEAs*, *DHN*, are induced by adverse conditions in most plants, and therefore are believed to participate in plant environmental stress tolerance^40^. Overexpression of *DHNs* improved salt, drought and cold tolerance in transgenic plants^41^,^42^,^43^,^44^.Therefore, LEA proteins are quite important in plant stress tolerance. Given their importance in stress tolerance, we further studied whether *BpERF11* alters the expression of *LEAs*. Three *LEA* family genes from birch, which are homologous to the *LEA* or *DHN* genes that were shown previously to be specifically involved in stress tolerance to salt and osmotic were selected to determine whether they are regulated by *BpERF11*^35^,^36^,^37^. The results showed that *BpERF11* could downregulate the expression of these genes (Fig. 8), suggesting that overexpression of *BpERF11* reduced the expression of *LEA* genes related to stress tolerance, and the reduction of these *LEAs* and *DHNs* might contribute to increase abiotic stress sensitivity.

### *BpERF11* reduces proline accumulation under salt and osmotic stress conditions

Proline is the main compatible solute in plants, and plays an important role in osmotic potential adjustment. Accumulation of proline can reduce cellular water potential to maintain the hydrological balance and avoid deleterious toxicity of high ionic strength. Proline also binds to proteins to serve as molecular chaperone to stabilize them^45^, and acts as a radical scavenger to scavenge intracellular ROS and inhibit ROS-mediated apoptosis^46^. The metabolism of proline also provides signaling ROS to regulate redox homeostasis and epigenetic reprogramming. Additionally, proline could maintain sustainable growth, and its homeostasis is involved in actively dividing cells under long-term stress^47^. In the metabolism of proline, P5CS is the key and rate limiting enzyme involved in the biosynthesis of proline, and PRODH and P5CDH are the two main enzyme for the degradation of proline^48^,^49^. Our study showed that the OE lines had lowest proline levels and the Ri lines showed increased proline levels, indicating that the transcript level of *BpERF11* negatively correlates with proline accumulation. Additionally, qRT-PCR showed that *BpERF11* in plants downregulates the expression of proline biosynthesis genes, but upregulates the expression of the two proline degradation genes (Fig. 6B,C). These results suggested that overexpression of *BpERF11* decreased salt and osmotic stress by reducing proline accumulation in plants, reflecting *BpERF11*’*s* ability to upregulate the expression of proline degradation genes and to downregulate the expression of proline biosynthesis genes.

### Overexpression of *BpERF11* reduces ROS scavenging capacity

When plants are exposed to adverse environments, such as salt, drought and low temperatures, ROS will accumulate rapidly, which damage cellular macromolecules and cellular membranes, and cause DNA damage. However, low levels of ROS function as signaling molecules in abiotic stress^50^,^51^. Therefore, modulation of ROS is critical for the abiotic stress tolerance. Previous studies showed that *ERFs* are involved in ROS scavenging. For instance, *Arabidopsis ERF6* serves as a positive antioxidant regulator to play an important role in plant in response to abiotic stresses^52^. Given the importance of ROS in the stress response, we determined ROS levels. DAB and NBT staining and H_2_O_2_ detection all showed that overexpression of *BpERF11* caused high accumulation of ROS. By contrast, knockdown of *BpERF11* significantly reduced ROS accumulation (Fig. 5). As SOD and POD are the main enzymes involved in ROS scavenging, we further checked the expression of *SOD* and *POD*s. The birch genes that are homologue to the Arabidopsis genes having SOD and POD activities were used in study. The results showed that *BpERF11* significantly dwonregulated the expression of *SOD* and *POD* genes (Fig. 5G,H). Taken together, these results indicated that *BpERF11* inhibits the expression of *SOD*s and *POD*s to decrease SOD and POD activities, which reduced the ROS scavenging capability leading to sensitivity to salt and osmotic stress.

## Conclusion

**Figure 9:**
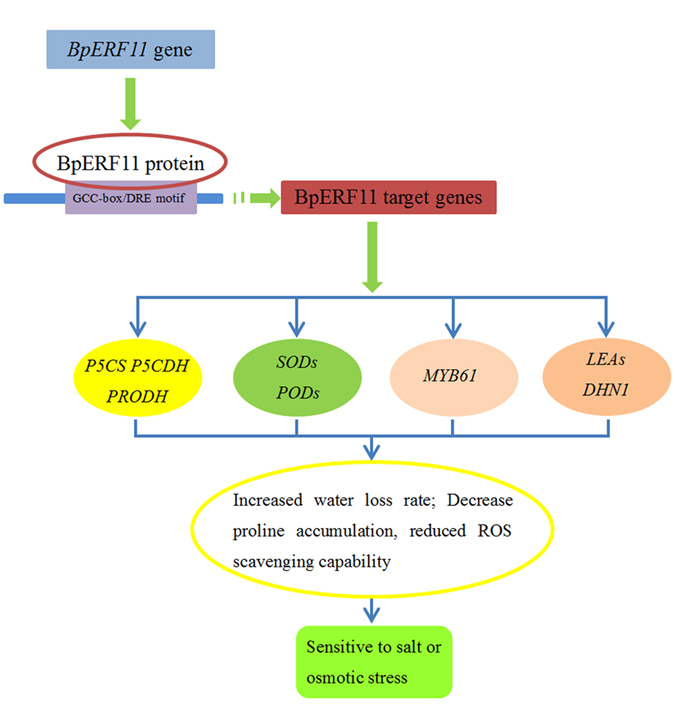
Model of the regulatory network of *BpERF11* in response to salt and severe osmotic stress. BpERF11 binds to the GCG-box or DRE motifs to regulate the expression of its target genes, such as *SODs*, *PODs*, *P5CS*, *P5CDH*, *PRODH*, *MYB61*, *DHN* and *LEAs*, which result in significant physiological changes, including decreased proline accumulation, reduced ROS scavenging capability, increased stomatal aperture, elevated MDA content, enhanced cell damage and reduced chlorophyll. These changes finally lead to increased sensitivity to salt and osmotic stress.

In the present study, we cloned an *ERF* gene, *BpERF11*, from birch, and observed that it could specifically bind to GCC boxes and DRE motifs to serve as a transcription factor. Our studies suggested that *BpERF11* modulates abiotic stress sensitivity at least in three ways: negative control of stomatal apertures leading to increase water loss rate; decreasing proline accumulation to reduce osmotic potential, and reducing ROS scavenging capability causing ROS damage. Additionally, our studies showed that *BpERF11* could regulate the expression of a series of stress-related genes including *PRODH*, *P5CDH*, *P5CS*, *DHN*, *LEA*, *MYB61*, *SODs* and *POD*s to induce the above physiological changes (Fig. 9).

## Methods

### Materials

*B. platyphylla* seedlings were grown in pots containing a mixture of perlite/soil (2:1) in a greenhouse under the conditions of 16/8 h light/dark, 70–75% relative humidity at 25 °C. Ten-week-old birch plants were watered with a solution of 200 mM NaCl, 200 mM mannitol or 20% polyethylene glycol (PEG6000) in their roots for 3, 6, 12, 24 and 48 h. The roots and leaves were harvested for RNA isolation. Plants watered with fresh water were harvested at the corresponding time points as controls. The leaves from the second node to apical leaves of birch were harvested, and were pooled together. The leaves or roots were pooled from at least 6 seedlings for each sample, and three independent biological replications were performed. Tissue cultured seedlings were cultivated in a tissue culture room with 70–75% relative humidity, a 12/12 h light/dark photoperiod and an average temperature of 24 °C.

### Gene cloning and sequence analysis of *BpERF11*

The full-length cDNA of *BpERF11* (GenBank ID: KT601336), was cloned from the birch transcriptome^34^. Multiple sequence alignments of BpERF11 with other ERFs from *Arabidopsis* were performed using ClustalW (http://www.ebi.ac.uk/clustalw/). A phylogenetic tree was constructed using MEGA5.1 with the neighbor-joining (NJ) method. The internal branch support was estimated with 1000 bootstrap replicates.

### Vector construction

The coding sequence (CDS) of *BpERF11*, without its stop codon, was PCR amplified and fused in frame to the N-terminus of the green fluorescent protein (GFP) gene under the control of a CaMV 35S promoter to generate the 35S:BpERF11-GFP construct. The CDS of *BpERF11* was also cloned into pROKII under the control of the CaMV 35S promoter (35S:BpERF11). To construct the knockdown vector of *BpERF11*, a 246 bp region of the promoter sequence of *BpERF11* was inserted into pFGC5941 in the forward and reverse directions to form an inverted repeat truncated promoter, separated by the ChsA intron (pFGC5941-BpERF11); all the primer sequences are shown in Table S1.

### Real-time qRT-PCR

Total RNA was isolated using the cetyltrimethyl bromide (CTAB) method^53^, and treated with DNaseI to remove DNA contamination. Total RNA (5 μg) was reverse-transcribed into cDNA using a PrimeScriptTM RT Reagent Kit (Takara Corp., Dalian, China) with oligo(dT) as the primer. The synthesized cDNA was diluted to 100 μL using water to use as real-time PCR template. Real-time PCR was performed on an MJ Opticon^TM2^ instrument (Bio-Rad, Hercules, CA, USA) with *Tubulin* (GenBank number: FG067376) and *Ubiquitin* (GenBank number: FG065618) as internal references. The conditions of real-time PCR were as follows: 2 μL of cDNA template (equivalent to 100 ng of total RNA), 0.5 μM forward and reverse primers respectively), 10 μL of SYBR Green Realtime PCR Master Mix (Toyobo) in a volume of 20 μL. The amplification conditions were as follows: 94 °C for 30 s; 45 cycles of 94 °C for 12 s, 58 °C for 30 s, 72 °C for 45 s; and 1 s at 81 °C for plate reading. Expression levels were calculated from the threshold cycle according to the delta-delta CT method^54^. Three biological replications were conducted. The primers used and the GenBank number of studied genes are listed in Table S2.

### Subcellular localization of BpERF11 protein

The 35S:BpERF11-GFP construct and 35S:GFP (control) were transformed separately into onion epidermal cells using particle bombardment (BioRad). After incubation on MS medium for 48 h in the dark, the onion epidermal cells were visualized under an LSM700 confocal laser microscope (Zeiss, Jena, Germany).

### Plant transformation

The 35S:BpERF11 and pFGC5941-BpERF11 were introduced separately into birch plants using an Agrobacterium tumefaciens-mediated transformation method. Briefly, birch leaves were cut into small pieces and soaked in Agrobacterium cell suspension culture (OD_600_ = 0.6) for 5 minutes, before being placed on woody plant medium (WPM + 2% (w/v) sucrose, pH 5.8) for 48 h. After co-culture, the plantlets were cultured in a selection medium (WPM + 1.0 mg·L^−1^ 6-BA + 2% (w/v) sucrose + 500 mg·L^−1^ carbenicillin) + 50 mg·L^−1^ kanamycin (for 35S:BpERF11 transformation)/2 mg·L^−1^ glyphosate (for pFGC5941-BpERF11 transformation) to induce resistant callus at 25 °C. The antibiotic-resistant calli were regenerated and transferred into differentiation medium (WPM + 1.0 mg·L^−1^ 6-BA + 50 mg·L^−1^ kanamycin/2 mg·L^−1^ glyphosate) for inducing bud differentiation. The adventitious buds were then moved to root induction medium (WPM + 0.2 mg·L^−1^ NAA + 50 mg·L^−1^ kanamycin (for 35S:BpERF11 transformation)/2 mg·L^−1^ glyphosate (for pFGC5941-BpERF11 transformation)). The expression level of *BpERF11* in transgenic birch lines was analyzed by qRT-PCR. According to the qRT-PCR, two overexpression lines (OE3, OE6) with high expression levels, and two RNA interference lines (Ri2, Ri3) with the most downregulated *BpERF11* expression levels were selected for further study.

### Analysis of growth phenotype

OE, Ri and WT plant lines of similar sizes were transferred to WPM medium supplied with 50 mM NaCl or 80 mM mannitol for 4 weeks; plants growing in WPM medium were used as controls. The plant height, root length, root numbers, fresh weight and dry weight were measured. Three biological replicates were analyzed for each experiment, and each experiment contained at least five plantlets.

### Histochemical analysis of plants in response to stress

Histochemical staining was performed to detect cell death and ROS. The OE, Ri and WT plantlets were treated with NaCl (150 mM) or mannitol (200 mM) for 1 and 2 h. Their leaves were detached immediately for histochemical staining. To detect superoxide and hydrogen peroxide *in situ*, leaves were infiltrated with nitroblue tetrazolium (NBT) or 3, 3′-diaminobenzidine (DAB) solutions, respectively. Cell death was determined by Evans blue staining. The staining process was performed according to Zhang *et al.* and Yang *et al.*^51^,^55^.

### Analysis of the physiological parameters related to stress tolerance

To determine the physiological effects on OE, Ri and WT lines in response to abiotic stress, OE, Ri and WT plantlets of similar sizes were transferred into pots containing a mixture of turf peat and sand (2:1 v/v). For salt or severe osmotic stress, they were treated with 150 mM NaCl or 200 mM mannitol, respectively, for 3 d. Plants watered with fresh water were used as the control. Superoxide dismutase (SOD) and peroxidase POD activities, the MDA content and relative electrical conductivity were measured according to Wang *et al.*^56^; chlorophyll and H_2_O_2_ contents were measured according to Minotti *et al.*^57^. Proline contents were determined according to Bates *et al.*^58^. Each sample contained at least 12 plantlets, and three biological replications were performed.

To measure the water loss rate, the fresh weights (FW) of leaves detached from WT, OE and Ri every 0.5 h for 7 h were weighed. The leaves were then dried overnight at 80 °C, and the dry weights (DW) were determined. The water loss rates were calculated using the formula: WC (%) = ((FW − desiccated weight)/(FW − DW)) × 100.

To measure the stomatal apertures, the epidermal peels were stripped from the leaves of WT, OE and Ri lines and floated in a solution of 30 mM KCl and 10 mM MES-KOH (pH 6.15), followed by incubation for 2 h in the light at 22 °C to induce stomatal opening^59^. After inducing stomatal opening, 50 mM NaCl or 100 mM mannitol was added to the buffer solution, respectively, and the samples were further incubated for 2 h. Stomatal apertures were photographed under a light microscope (Olympus BX43, Japan). Measurements were performed using the free software IMAGEJ 1.36b (Broken Symmetry Software; http://brokensymmetry.com).

### Yeast one-hybrid (Y1H) analysis

Y1H was performed to determine the binding of BpERF11 to GCC boxes and DRE motifs (Clontech, Palo Alta, CA, USA). Three tandem copies of the GCC box (AGCCGCC), DRE (TACCGACAT) or their mutants, GCC-m1 (AGTTGCC), GCC-m2 (ATCCTCC), GCC-m3 (TTTTTTT), DRE-m1 (TATTGACAT), DRE-m2 (TACCTTCAT) or DRE-m3 (TTTTTTTTT) were cloned separately into pHIS2 as reporters. *BpERF11* was cloned into pGADT7-rec2 as the effector vector. The effector, together with each reporter, was co-transformed into Y187 for Y1H analysis. The primers used are listed in Table S3.

### Expression levels of target genes of *BpERF11* assessed by qRT-PCR analysis

OE, Ri and WT birch plantlets were treated with 150 mM NaCl or 200 mM mannitol for 72 h and harvested for qRT-PCR analysis. Plants watered with fresh water only were harvested at the same time and used as controls. The expression of *SOD*, *POD*, Δ1-pyrroline-5-carboxylate synthetase (*BpP5CS*), Pro-dehydrogenase (*BpPRODH*), P5C-dehydrogenase (*BpP5CDH*), stomatal aperture control gene (*BpMYB61*), and late embryogenesis abundant protein genes (*LEA1*, *LEA2* and *DHN1*) were analyzed using qRT-PCR. Three biological replicates were performed. The primers used are listed in Table S4.

### Statistical analyses

Statistical analyses were performed using the SPSS software package (SPSS, Chicago, Illinois, USA). One-way ANOVA was used to determine the statistical significance of the results, and the differences were considered statistically significant at P < 0.05. The error bar represents the standard deviation (S. D.).

## Additional Information

**How to cite this article**: Zhang, W. *et al.* An Ethylene-responsive Factor *BpERF11* Negatively Modulates Salt and Osmotic Tolerance in *Betula platyphylla*. *Sci. Rep.* **6**, 23085; doi: 10.1038/srep23085 (2016).

## Supplementary Material

Supplementary Information

## References

[b1] Wang W., Vinocur B. & Altman A. Plant responses to drought, salinity and extreme temperatures: towards genetic engineering for stress tolerance. Planta 218, 1–14 (2003). 14513379 10.1007/s00425-003-1105-5

[b2] Burke E. J., Brown S. J. & Christidis N. Modelling the recent evolution of global drought and projections for the twenty-first century with the hadley centre climate model. J. Hydrometeor 7, 1113–1125 (2006).

[b3] Agarwal P. K., Agarwal P., Reddy M. K. & Sopory S. K. Role of DREB transcription factors in abiotic and biotic stress tolerance in plants. Plant Cell Rep. 25, 1263–1274 (2006). 16858552 10.1007/s00299-006-0204-8

[b4] Riechmann J. L. *et al.* *Arabidopsis* transcription factors: genome-wide comparative analysis among eukaryotes. Science 290, 2105–2110 (2000). 11118137 10.1126/science.290.5499.2105

[b5] Guo A. *et al.* DATF: a database of *Arabidopsis* transcription factors. Bioinformatics 21, 2568–2569 (2005). 15731212 10.1093/bioinformatics/bti334

[b6] Iida K. *et al.* RARTF: database and tools for complete sets of *Arabidopsis* transcription factors. DNA Res. 12, 247–256 (2005). 16769687 10.1093/dnares/dsi011

[b7] Century K., Reuber T. L. & Ratcliffe O. J. Regulating the regulators: the future prospects for transcription-factor-based agricultural biotechnology products. Plant Physiology 147, 20–29 (2008). 18443103 10.1104/pp.108.117887 PMC2330319

[b8] Kizis D., Lumbreras V. & Pages M. Role of AP2/EREBP transcription factors in gene regulation during abiotic stress. FEBS Lett. 498, 187–189 (2001). 11412854 10.1016/s0014-5793(01)02460-7

[b9] Sakuma Y., Liu Q. & Dubouzet J. G. DNA-binding specificity of the ERF/AP2 domain of *Arabidopsis* DREBs, transcription factors involved in dehydration-and cold-inducible gene expression. Biochem Biophys Res Commun. 290, 998–1009 (2002). 11798174 10.1006/bbrc.2001.6299

[b10] Tang W., Charles T. M. & Newton R. J. Overexpression of the pepper transcription factor *CaPF1* in transgenic *Virginia pine* (*Pinus virginiana* Mill.) confers multiple stress tolerance and enhances organ growth. Plant Mol Biol. 59, 603–617 (2005). 16244910 10.1007/s11103-005-0451-z

[b11] Gutterson N. & Reuber T. L. Regulation of disease resistance pathways by AP2/ERF transcription factors. Curr Opin Plant Biol. 7, 465–471 (2007). 15231271 10.1016/j.pbi.2004.04.007

[b12] Jofuku K. D., Denboer, B., G. W. & Vanmontagu M. Control of *Arabidopsis* Flower and seed Development by the Homeotic Gene APETA-LA2. Plant Cell 6, 1211–1225 (1994). 7919989 10.1105/tpc.6.9.1211 PMC160514

[b13] Nakano T. *et al.* Genome-Wide Analysis of the ERF Gene Family in *Arabidopsis* and Rice. Plant physio. 140, 411–432 (2006). 10.1104/pp.105.073783 PMC1361313 16407444

[b14] Zhang G. *et al.* Phylogeny, gene structures, and expression patterns of the ERF gene family in soybean (*Glycine max L.*). J Exp Bot. 59, 4095–4107 (2008). 18832187 10.1093/jxb/ern248 PMC2639015

[b15] Buttner M. *et al.* *Arabidopsis thaliana* ethylene-responsive element binding protein (AtEBP), an ethylene-inducible, GCC box DNA binding protein interacts with an ocs element binding protein. Proc Natl Acad Sci USA 94, 5961–5966 (1997). 9159183 10.1073/pnas.94.11.5961 PMC20889

[b16] Lee J. H. *et al.* The ethylene-responsive factor like protein 1(CaERFLP1) of hot pepper (*Capsicum annuum L*.) interacts *in vitro* with both GCC and DRE CRT sequences with different binding affinities: Possible biological roles of *CaERFLP1* in response to pathogen infection and high salinity conditions in transgenic tobacco plants. Plant Mol Bio. 55, 61–81 (2004). 15604665 10.1007/s11103-004-0417-6

[b17] Zhang H. W. *et al.* The ethylene-, jasmonate-, abscisic acid- and NaCl-responsive tomato transcription factor *JERF1* modulates expression of GCC box-containing genes and salt tolerance in tobacco. Planta 220, 262–270 (2004). 15300440 10.1007/s00425-004-1347-x

[b18] Zhang G. *et al.* Overexpression of the soybean *GmERF3* gene, an AP2/ERF type transcription factor for increased tolerances to salt, drought, and diseases in transgenic tobacco. J Exp Bot. 60, 3781–3796 (2009). 19602544 10.1093/jxb/erp214 PMC2736888

[b19] Dey S. & Corina V. A. Ethylene responsive factors in the orchestration of stress responses in monocotyledonous plants. Front Plant Sci, 10.3389/fpls.2015.00640 (2015). PMC4552142 26379679

[b20] Licausi F., Ohme T. M. & Perata P. APETALA2/Ethylene Responsive Factor (AP2/ERF) transcription factors: mediators of stress responses and developmental programs. New Phytol. 199, 639–49 (2013). 24010138 10.1111/nph.12291

[b21] Riechmann J. L. & Meyerowitz E. M. The AP2/EREBP family of plant transcription factors. Biol Chem. 379, 633–646 (1998). 9687012 10.1515/bchm.1998.379.6.633

[b22] Nakano T. *et al.* The AP2/ERF transcription factor *SlERF52* functions in flower pedicel abscission in tomato. J Exp Bot. 65, 3111–3119 (2014). 24744429 10.1093/jxb/eru154 PMC4071829

[b23] Lee S. Y. *et al.* *Arabidopsis AtERF71/HRE2* functions as transcriptional activator via cis-acting GCC box or DRE/CRT element and is involved in root development through regulation of root cell expansion. Plant Cell Rep. 34, 223–31 (2015). 25344007 10.1007/s00299-014-1701-9

[b24] Xu Z. *et al.* Isolation and molecular characterization of the *Triticum aestivum L.* ethylene-responsive factor 1 (*TaERF1*) that increases multiple stress tolerance. Plant Mol Biol. 65, 719–732 (2007). 17874224 10.1007/s11103-007-9237-9

[b25] Mishra S. *et al.* *PsAP2* an AP2/ERF family transcription factor from *Papaver somniferum* enhances abiotic and biotic stress tolerance in transgenic tobacco. Plant Mol Biol. 89, 173–86 (2015). 26319514 10.1007/s11103-015-0361-7

[b26] Gao S., Zhang H. & Tian Y. Expression of *TERF1* in rice regulates expression of stress-responsive genes and enhances tolerance to drought and high-salinity. Plant Cell Rep. 27, 1787–1795 (2008). 18777179 10.1007/s00299-008-0602-1

[b27] Park H. Y., Seok H. Y., Woo D. H. & Lee S. Y. *AtERF71/HRE2* transcription factor mediates osmotic stress response as well as hypoxia response in *Arabidopsis*. Biochem Biophys Res Commun 414, 135–141 (2011). 21946064 10.1016/j.bbrc.2011.09.039

[b28] Zhai Y. *et al.* Isolation and molecular characterization of *GmERF7*, a soybean ethylene-response factor that increases salt stress tolerance in tobacco. Gene 513, 174–183 (2013). 23111158 10.1016/j.gene.2012.10.018

[b29] Yang Z. *et al.* *Arabidopsis ERF4* is a transcriptional repressor capable of modulating ethylene and abscisic acid responses. Plant Mol Biol. 58, 585–596 (2005). 16021341 10.1007/s11103-005-7294-5

[b30] Song C. P. *et al.* Role of an *Arabidopsis* AP2/EREBP-type transcriptional repressor in abscisic acid and drought stress responses. Plant Cell 17, 2384–2396 (2005). 15994908 10.1105/tpc.105.033043 PMC1182496

[b31] Liu D. F. *et al.* The rice ERF transcription factor *OsERF922* negatively regulates resistance to *Magnaporthe oryzae* and salt tolerance. J Exp Bot. 63, 3899–3912 (2012). 22442415 10.1093/jxb/ers079 PMC3388842

[b32] Tian Z. *et al.* The potato ERF transcription factor *StERF3* negatively regulates resistance to phytophthora infestans and salt tolerance in potato. Plant Cell Physiol. 56, 992–1005 (2015). 25681825 10.1093/pcp/pcv025

[b33] Dubois M. *et al.* The ethylene response factors *ERF6* and *ERF11* antagonistically regulate mannitol-induced growth inhibition in *Arabidopsis*. Plant Physiol. 169, 166–179 (2015). 25995327 10.1104/pp.15.00335 PMC4577380

[b34] Wang C. *et al.* Comprehensive transcriptome analysis of developing xylem responding to artificial bending and gravitational stimuli in *Betula platyphylla*. PloS one 9, e87566 (2014). 24586282 10.1371/journal.pone.0087566 PMC3930542

[b35] Jia F. *et al.* Overexpression of Late Embryogenesis Abundant 14 enhances Arabidopsis salt stress tolerance. Biochem Biophys Res Commun. 454, 505–511 (2014). 25450686 10.1016/j.bbrc.2014.10.136

[b36] Zhao P. *et al.* Overexpression of *AtLEA3-3* confers resistance to cold stress in Escherichia coli and provides enhanced osmotic stress tolerance and ABA sensitivity in *Arabidopsis thaliana*. Mol Biol (Mosk). 45, 851–862 (2011). 22393782

[b37] Shi H., Ye T., Yang F. & Chan Z. *Arabidopsis PED2* positively modulates plant drought stress resistance. J Integr Plant Biol. 10.1111/jipb.12330 (2015). 25588806

[b38] Liang Y. K. *et al.* *AtMYB61*, an R2R3-MYB transcription factor controlling stomatal aperture in *Arabidopsis thaliana*. Curr Biol. 15, 1201–1206 (2005). 16005292 10.1016/j.cub.2005.06.041

[b39] Serrano R. & Montesinos C. Molecular bases of desiccation tolerance in plant cells and potential applications in food dehydration. Food Sci Technol Int. 9, 157–161 (2003).

[b40] Yang W. *et al.* The K-segments of wheat dehydrin WZY2 are essential for its protective functions under temperature stress. Front Plant Sci, 10.3389/fpls.2015.00406 (2015). PMC4467595 26124763

[b41] Vítámvás P. *et al.* Quantitative analysis of proteome extracted from barley crowns grown under different drought conditions. Front Plant Sci, 10.3389/fpls.2015.00479 (2015). PMC4485253 26175745

[b42] Chiappetta A. *et al.* A dehydrin gene isolated from feral olive enhances drought tolerance in *Arabidopsis* transgenic plants. *Front* Plant Sci. 30, 392 (2015). 10.3389/fpls.2015.00392 PMC4485055 26175736

[b43] Saibi W., Feki K., Ben M. R. & Brini F. Durum wheat dehydrin (*DHN-5*) confers salinity tolerance to transgenic *Arabidopsis* plants through the regulation of proline metabolism and ROS scavenging system. Planta 242, 1187–1194 (2015). 26105651 10.1007/s00425-015-2351-z

[b44] Byun M. Y. *et al.* Constitutive expression of *DaCBF7*, an Antarctic vascular plant Deschampsia antarctica CBF homolog, resulted in improved cold tolerance in transgenic rice plants. Plant Sci. 236, 61–74 (2015). 26025521 10.1016/j.plantsci.2015.03.020

[b45] Maggio A. *et al.* Does proline accumulation play an active role in stress-induced growth reduction? Plant J. 31, 699–712 (2002). 12220262 10.1046/j.1365-313x.2002.01389.x

[b46] Chen C. & Dickman M. B. Proline suppresses apoptosis in the fungal pathogen Colletotrichum trifolii. Proc Natl Acad Sci USA 102, 3459–3464 (2005). 15699356 10.1073/pnas.0407960102 PMC552905

[b47] Kavi Kishor P. B. & Sreenivasulu N. Is proline accumulation per se correlated with stress tolerance or is proline homeostasis a more critical issue? Plant Cell Environ. 37, 300–311 (2013). 23790054 10.1111/pce.12157

[b48] Silva-Ortega C. O. *et al.* Salt stress increases the expression of p5cs gene and induces proline accumulation in cactus pear. Plant Physiol Biochem. 46, 82–92 (2008). 18054243 10.1016/j.plaphy.2007.10.011

[b49] Chen J. B. *et al.* Two P5CS genes from common bean exhibiting different tolerance to salt stress in transgenic Arabidopsis. J Genet. 92, 461–479 (2013). 24371167 10.1007/s12041-013-0292-5

[b50] Wang F. Z. *et al.* Enhanced drought tolerance of transgenic rice plants expressing a pea manganese superoxide dismutase. J Plant Physiol. 162, 465–472 (2005). 15900889 10.1016/j.jplph.2004.09.009

[b51] Zhang X. *et al.* Maize ABP9 enhances tolerance to multiple stresses in transgenic *Arabidopsis* by modulating ABA signaling and cellular levels of reactive oxygen species. Plant Mol Biol. 75, 365–378 (2011). 21327835 10.1007/s11103-011-9732-x PMC3044229

[b52] Sewelam N. *et al.* Ethylene response factor 6 is a regulator of reactive oxygen species signaling in *Arabidopsis*. PLoS One 8, e70289 (2013). 23940555 10.1371/journal.pone.0070289 PMC3734174

[b53] Chang S., Puryear J. & Cairney J. A simple and efficient method for isolating RNA from pine trees. Plant Mol Biol Rep. 11, 113–116 (1993).

[b54] Livak K. J. & Schmittgen T. D. Analysis of relative gene expression data using real-time quantitative PCR and the 2(-Delta Delta C(T)) Method. Methods 25, 402–408 (2001). 11846609 10.1006/meth.2001.1262

[b55] Yang G. Y. *et al.* Overexpression of a GST gene (*ThGSTZ1*) from *Tamarix hispida* improves drought and salinity tolerance by enhancing the ability to scavenge reactive oxygen species. Plant Cell Tissue Organ Culture. 117, 99–112 (2014).

[b56] Wang Y. *et al.* A novel bZIP gene from *Tamarix hispida* mediates physiological responses to salt stress in tobacco plants. J Plant Physiol. 167, 222–230 (2010). 19853962 10.1016/j.jplph.2009.09.008

[b57] Minotti P., Halseth D. & Sieczka J. Field chlorophyll measurements to assess the nitrogen status of potato varieties. HortScience 29, 1497–1500 (1994).

[b58] Bates L. S., Waldren R. P. & Teare J. D. Rapid determination of free proline for water stress studies. Plant Soil. 39, 205–207 (1973).

[b59] Cheng M. C. *et al.* The *Arabidopsis* ethylene response factor1 regulates abiotic stress-responsive gene expression by binding to different cis-acting elements in response to different stress signals. Plant physiol. 162, 1566–1582 (2013). 23719892 10.1104/pp.113.221911 PMC3707555

