# Supplementary material for: An Ethylene-responsive Factor BpERF11 Negatively Modulates Salt and Osmotic Tolerance in Betula platyphylla
Source: Sci Rep. 2016 Mar 16;6:23085. doi: 10.1038/srep23085 (PMC4793294; doi:10.1038/srep23085)
Supplement: Supplementary Information [file srep23085-s1.doc]

**An Ethylene-responsive Factor *BpERF11* Negatively Modulates Salt and Osmotic Tolerance in *Betula platyphylla***

Wenhui Zhang^1, 2^, Guiyan Yang^1^, Dan Mu^1^, Hongyan Li^1^, Dandan Zang^1^, Hongyun Xu^1^, Xuezhong Zou^1, 3^, Yucheng Wang^1^*

^1^ State Key Laboratory of Forest Genetics and Tree Breeding, Northeast Forestry University, 150040 Harbin, China

^2^ Agronomy College, Heilongjiang Bayi Agricultural University, 163319 Daqing, China

^3^ Liaoning Forestry Vocation-Technical College, 110101 Shenyang, China

*Corresponding author: Yucheng Wang

E-mail: [wangyucheng@ms.xjb.ac.cn](mailto:wangyucheng@ms.xjb.ac.cn)

Tel: +86-451-82190607-15

Fax: +86-451-82190607-11

**Fig. S1** Sequence alignments and phylogenetic analyses of BpERF11 and ERFs from *A. thaliana*. A. Alignments of BpERF11 with *Arabidopsis thaliana* ERFs. Red boxes marks the V and E conserved amino acid residues at positions 14 and 19; B. Phylogenetic tree of BpERF11 and ERFs from *A. thaliana*. All amino acid sequences were retrieved from the Tair database including AT3G50260.1(AtERF11), AT1G12610.1(DDF1), AT1G25470.1(CRF12), AT1G33760.1(AtERF22), AT1G44830.1(AtERF14), AT1G50640.1(AtERF3), AT1G80580.1, AT2G23340.1(DEAR3), AT2G31230.1(AtERF15), AT2G38340.1(DREB19), T2G44840.1(AtERF13), AT3G11020.1(DREB2), AT2G47520.1(AtERF71), AT3G16770.1(AtERF72), AT3G20310.1(AtERF7), AT4G11140.1(CRF1), AT3G61630.1(CRF6), T4G11140.1(CRF1), AT4G25480.1(CBF3), AT5G44210.1(AtERF9), AT5G50080.1(AtERF110) and AT5G51990.1(CBF4).


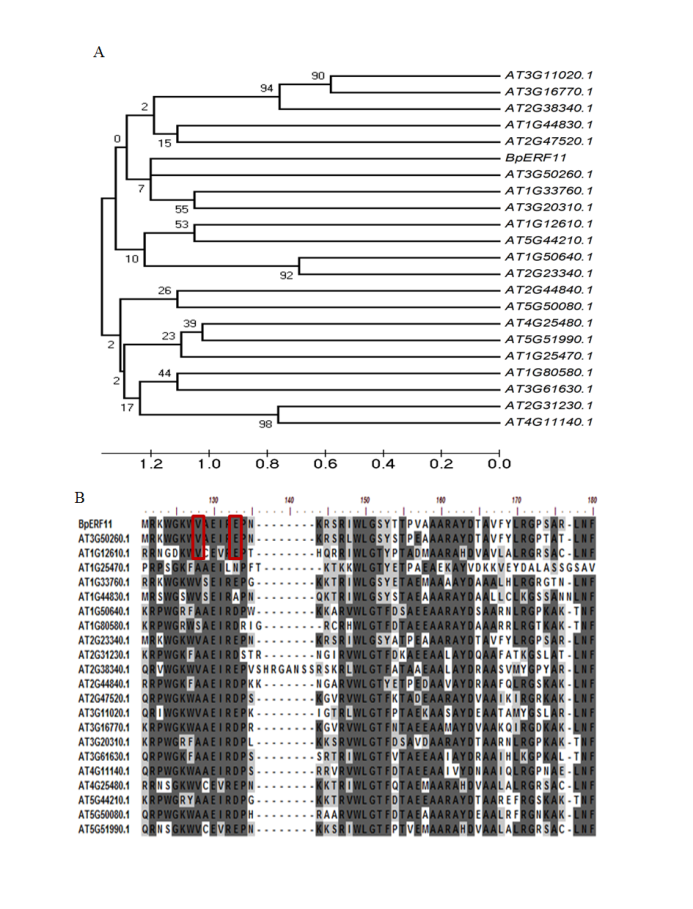


**Fig. S2** The expression of *BpERF11* in the transgenic birch plants overexpressing *BpERF11*or RNAi-silenced *BpERF11*. The relative expression level was normalized by the expression level in WT plants and log2 transformed. Three biological replications were conducted. The error bars represent the standard deviation (S. D.).


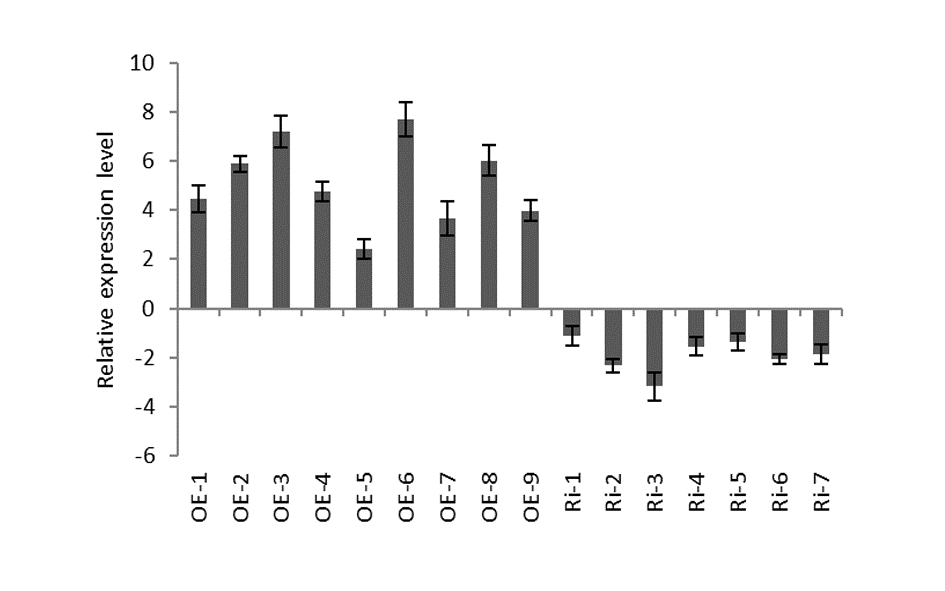


**Fig. S3** Analyses of chlorophyll content, electrolyte leakage and Evans blue staining. A. Chlorophyll content; B. Electrolyte leakage; The birch plantlets of OE, Ri and WT lines of similar sizes grown in a mixture of turf peat and sand were treated with 150 mM NaCl or 200 mM mannitol for 3 d. Plants watered with fresh water were used as the control. C. Evans blue staining. Cell death was determined by Evans blue staining, the plantlets of OE, Ri and WT were treated with NaCl (150 mM) or mannitol (200 mM) for 1 and 2 h, their leaves were detached immediately for Evans blue staining. Three biological replications were conducted. The error bars represent the standard deviation (S. D.).


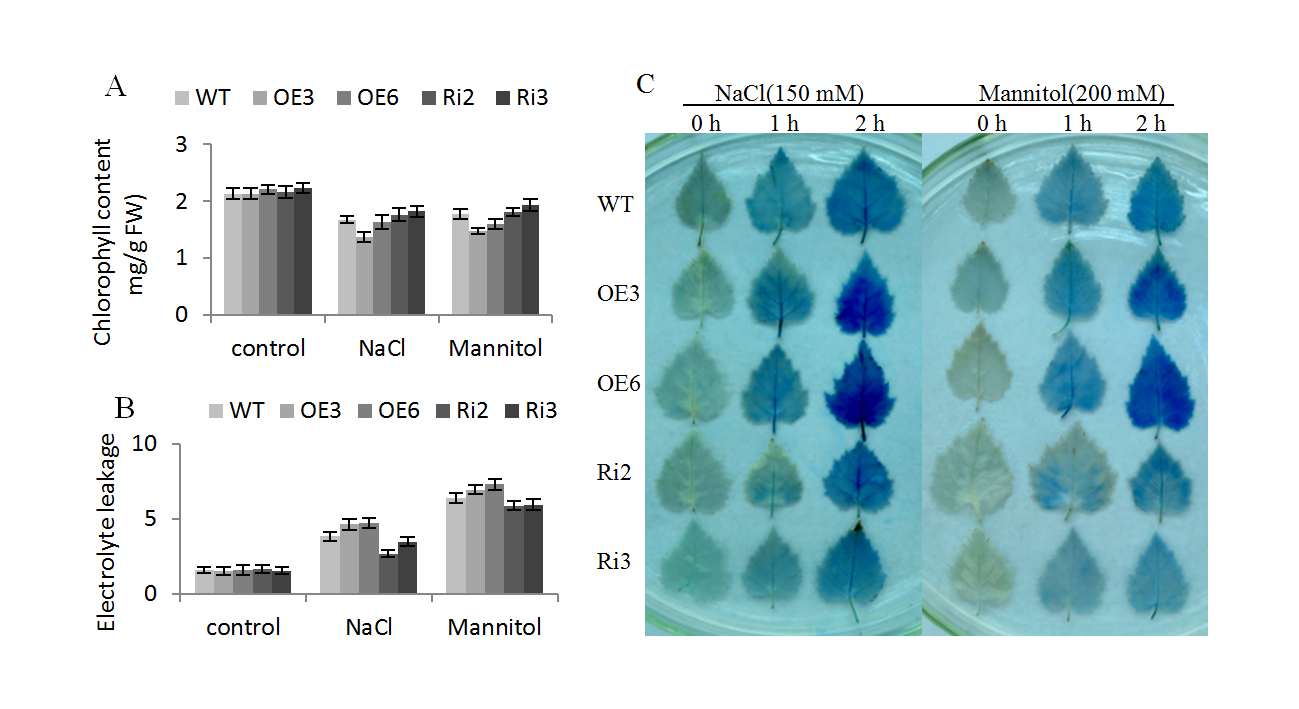


**Tables**

Table S1 The primers used for plasmid construction

| Primer | Sequence (5’-3’) | |
| --- | --- | --- |
| BpERF11-F | | GGTTGGATCCATGGAAGGAGACTGTTATTCGTCG |
| BpERF11-R | | ATCTTCATCCGAGTTTTCCG |
| GFP-R | | TCGAGCTCGGTACCCTCACTTGTACAGCTCATCCATGCC |
| pROKII-F | | GGCGAACGTGGCGAGAAAGG |
| pROKII-R | | ACAGGTTTCCCGACTGGAAAGC |
| pROKII-BpERF11-F | | CGGGATCCATGGAAGGAGACTGTTATTC |
| pROKII-BpERF11- R | | GTCAGAGCTCTCAATCTTCATCCGAGTTTTC |
| pFGC-BpERF11-Cis-F | | CATGCCATGGCACTCTTTAGAGTTATTTGT |
| pFGC-BpERF11-Cis-R | | CTAGATTTAAATAGGAGGCGGGGCAAATTAGTT |
| pFGC-BpERF11-Anti-F | | GCTCTAGACACTCTTTAGAGTTATTTGT |
| pFGC-BpERF11-Anti-R | | CGGGATCCAGGAGGCGGGGCAAATTAGTT |

Table S2 The primers of *BpERF11* used for real time RT-PCR

| Genes | GenBank number | Forward Primers (5’-3’) | Reverse Primers (5’-3’) |
| --- | --- | --- | --- |
| BpERF11 | KT601336 | TCAGGTGGAGGTGAGAAA | CAAGAAGGGAGTGCAAAT |
| Tubulin | FG067376 | TCAACCGCCTTGTCTCTCAGG | TGGCTCGAATGCACTGTTGG |
| Ubiquitin | FG065618 | GATTGAGGGGAGGGATGCTG | GGAGGACAAGGTGGAGGGTG |

Table S3 The sequences of primers used in Y1H assay

| Primer | Sequence (5’-3’) |
| --- | --- |
| BpERF11-AD-Rec2-F | TGGCCATTATGGCCCGGGATGGAAGGAGACTGTTATTC |
| BpERF11-AD-Rec2-R | GACATGTTTTTTCCCGGGTCAATCTTCATCCGAGTTTTC |
| pHIS2-F | GCCTTCGTTTATCTTGCCTGCTC |
| pHIS2-R | CGATCGGTGCGGGCCTCTTC |
| GCC-F | AATTCAGCCGCCAGCCGCCAGCCGCCGAGCT |
| GCC-R | CGGCGGCTGGCGGCTGGCGGCTG |
| GCC-M1-F | AATTCAGTTGCCAGTTGCCAGTTGCCGAGCT |
| GCC-M1-R | CGGCAACTGGCAACTGGCAACTG |
| GCC-M2-F | AATTCATCCTCCATCCTCCATCCTCCGAGCT |
| GCC-M2-R | CGGAGGATGGAGGATGGAGGATG |
| GCC-M3-F | AATTCTTTTTTTTTTTTTTTTTTTTTGAGCT |
| GCC-M3-R | CAAAAAAAAAAAAAAAAAAAAAG |
| DRE-F | AATTCTACCGACATTACCGACATTACCGACATGAGCT |
| DRE-R | CATGTCGGTAATGTCGGTAATGTCGGTAG |
| DRE-M1-F | AATTCTATTGACATTATTGACATTATTGACATGAGCT |
| DRE-M1-R | CATGTCAATAATGTCAATAATGTCAATAG |
| DRE-M2-F | AATTCTACCTTCATTACCTTCATTACCTTCATGAGCT |
| DRE-M2-R | CATGAAGGTAATGAAGGTAATGAAGGTAG |
| DRE-M3-F | AATTCTTTTTTTTTTTTTTTTTTTTTTTTTTTGAGCT |
| DRE-M3-R | CAAAAAAAAAAAAAAAAAAAAAAAAAAAG |

Table S4 Primers of relative genes used for real time RT-PCR

| Primers | GenBank number | Forward Primers (5’-3’) | Reverse Primers (5’-3’) |
| --- | --- | --- | --- |
| SOD1 | KP711288 | CCGTGGTTGTTCTTGGCAAC | CCAGCAGGATTGAAATGTGGC |
| SOD2 | KP711289 | TGCTCCTAAAGCGGTGGTTC | GGGTTGAAATGAGGGCCAGT |
| SOD3 | KP711290 | GAAGCCGCACTAGTAGCCAT | ACGACAGTGAGTGGTTTGGC |
| SOD4 | KP711291 | CGGAGGTCATATCAACCACTC | CAGACCAAGCCACACCCAT |
| SOD5 | KP711292 | ATGTAGGTCTGGTGGTGCTTC | AGAGAAGTGTGTGGATGGCTC |
| SOD6 | KP711293 | TGCTCCTAAAGCGGTGGTTC | GGGTTGAAATGAGGGCCAGT |
| POD1 | KP711296 | TCCGATCATGCTGCGTCTC | ATCGGCGTAGGAGAGGATGG |
| POD2 | KP711297 | ATTACCCAACTGTGAGCGAGG | CTGGATGCCTTATCGTCCCA |
| POD3 | KP711298 | CTGGTAAGGGTGACGAGGAC | GCTGGTAGTCAAAGCAGCATC |
| POD4 | KP711299 | GTGCAAGGAAGCTAAGTTGTT | TAGTCTCACAAGACCAGGAGC |
| POD5 | KP711300 | GTGCTGTTGGATGATACGGG | CTTTCCAAGAAGGACCACCC |
| POD6 | KP711301 | AAATGACCAAACAGTTCCAGCC | TCCACTGCTTTCTTTGCGTT |
| P5CS1 | KP711294 | CAAACGCCACCTCACAGAC | TCGTGTAACAACGGCAGTCC |
| P5CS2 | KP711295 | TGGGAACGGTCTACTGCTT | TTACTGCCTCTTGGGATTAC |
| PRODH | KP711308 | GAGTACACTTCCATCCAACC | ATGCTCCCCTCACCAAT |
| P5CDH | KP711306 | GACTTCAGCCTCCAACACCT | TCAACCAGCCTAACCAACC |
| LEA1 | KT601337 | GGCCAACATAGAGAAGCCAGA | ATCGTCCCCGATGCAATCA |
| LEA2 | KT601338 | AGTTATGAGACGGCCCAATC | CACCTGCTCTCCAGTCTTTTG |
| Hydrin1 | KT601339 | TGGTAACCCGATTCGAACAGA | AGCCCCTTCTTCTTCCTCCT |
| MYB61 | KT344120 | GAGGCCTGATTTGAAG | TGAACTTTCTGCCTTG |
| Tubulin | FG067376 | TCAACCGCCTTGTCTCTCAGG | TGGCTCGAATGCACTGTTGG |
| Ubiquitin | FG065618 | GATTGAGGGGAGGGATGCTG | GGAGGACAAGGTGGAGGGTG |
